# BRAF D594A mutation defines a unique biological and immuno-modulatory subgroup associated with functional CD8^+^ T cell infiltration in colorectal cancer

**DOI:** 10.1186/s12967-023-04606-5

**Published:** 2023-10-18

**Authors:** Wenjing Li, Chenyi Zhao, Wenhui Li, Yang Gong, Kaili Ma, Yujie Lu, Xiaowei Liu, Lianjun Zhang, Feng Guo

**Affiliations:** 1 Department of Clinical Laboratory, The Affiliated Suzhou Hospital of Nanjing Medical University, Suzhou Municipal Hospital, Gusu School, Nanjing Medical University, Suzhou, 215168 Jiangsu China; 2 National Key Laboratory of Immunity and Inflammation, Suzhou Institute of Systems Medicine, Chinese Academy of Medical Sciences & Peking Union Medical College, Suzhou, 215123 Jiangsu China; 3 Key Laboratory of Synthetic Biology Regulatory Elements, Suzhou Institute of Systems Medicine, Chinese Academy of Medical Sciences & Peking Union Medical College, Suzhou, 215123 Jiangsu China; 4 Department of Oncology, The Affiliated Suzhou Hospital of Nanjing Medical University, Suzhou Municipal Hospital, Gusu School, Nanjing Medical University, Suzhou, 215001 Jiangsu China

**Keywords:** Colorectal cancer, BRAF, D594A, THBS1, CXCL9/CXCL10

## Abstract

**Background:**

BRAF non-V600 mutation occupies a relatively small but critical subset in colorectal cancer (CRC). However, little is known about the biological functions and impacts of BRAF class III mutation in CRC. Here, we aim to explore how D594A mutation impacts on biological behaviors and immune related signatures in murine CRC cells.

**Methods:**

BRAF V600E (class I), G469V (class II) and D594A (class III) mutant cell lines were established based on MC38 cells. The biological behaviors of cells were evaluated in respect of cell growth, cell proliferation, cell apoptosis, cell migration and invasion by the methods of colony-forming assay, CCK-8 assay, Annexin V/PI staining and transwell assay. The concentrations of soluble cytokines were detected by ELISA. The membrane expression of immuno-modulatory molecules and the pattern of tumor infiltrating lymphocyte were evaluated by flow cytometry. The molecular mechanism was explored by RNA sequencing. Immunohistochemistry (IHC) staining was used for the detection of CD8α in tumor tissues. qRT-PCR and western blot were performed to assess the mRNA and protein expression. Anti-PD-L1 treatment and cytokines neutralization experiments were conducted in in vivo models.

**Results:**

D594A mutant cells displayed lower grade malignancy characteristics than V600E (class I) and G469V (class II) mutant cells. Meanwhile, D594A mutation led to evident immuno-modulatory features including upregulation of MHC Class I and PD-L1. In vivo experiments displayed that the frequency of infiltrated CD8^+^ T cells was significantly high within D594A mutant tumors, which may provide potential response to anti-PD-L1 therapy. RNA sequencing analysis showed that D594A mutation led to enhanced expression of ATF3 and THBS1, which thus facilitated CXCL9 and CXCL10 production upon IFN-γ treatment. In addition, CXCL9 or CXCL10 neutralization reduced the infiltration of CD8^+^ T cells into THBS1-overexpressing tumors.

**Conclusions:**

D594A mutant CRC exhibited lower aggressiveness and immune-activated phenotype. ATF3-THBS1-CXCL9/CXCL10 axis mediated functional CD8^+^ T cells infiltration into the microenvironment of D594A mutant CRC. Our present study is helpful to define this mutation in CRC and provide important insights in designing effective immunotherapeutic strategies in clinic.

**Supplementary Information:**

The online version contains supplementary material available at 10.1186/s12967-023-04606-5.

## Background

Mitogen-activated protein kinase (MAPK) cascade is involved in the progression of most human cancers, and mutations of RAS-RAF-MEK-ERK are frequently occurred. BRAF is an oncogenic kinase and contains diverse mutations in approximately 10% colorectal cancer (CRC) [1]. BRAF mutation can be classified as class I (V600D/E/K/R), class II (G469A/V/S), and class III (D594A/E/G/H/N/V) based on kinase activity, RAS-dependency and dimerization status [2].

Among the distinct BRAF mutations, the proportion of non-V600 (class II and class III) was approximately 22–30%, and V600 mutation is predominantly possessed in BRAF mutant CRC [2]. Recent retrospective studies revealed that non-V600 BRAF mutations exhibit a better prognosis than V600 mutations in metastatic CRC (mCRC) [1, 3]. Furthermore, the median of overall survival (OS) and progression free survival (PFS) of class III in CRC patients are significantly prolonged as compared to those of class I and II patients. However, the molecular mechanisms underlying those important clinical observations remain largely unknown [4].

Recently, immunotherapy emerged to represent the promising treatment modality for advanced CRC patients, including immune checkpoint inhibitors (ICIs), which acts largely within the tumor immune microenvironment (TIME) [5, 6]. Interestingly, among of BRAF wild type (WT) and mutation, TCGA (The Cancer Genome Atlas) and GEO (Gene Expression Omnibus) of CRC tissue specimens exhibited strikingly distinct infiltration pattern of diverse immune subsets within the TIME, with higher expression of immunoinhibitory molecules (PD-1, PD-L1, CTLA-4, LAG-3, and TIM3), enhanced immune cell infiltration (neutrophils and macrophages M1) and less tumor component in BRAF mutant tumors [7]. Unfortunately, ICIs achieve considerable therapeutic efficacy only in a small fraction of CRC patients whereas the majority of CRC patients develop primary or adaptive resistance to those ICI strategies. Tumor infiltrated CD8^+^ T cells, the main player of anti-tumor immunity and producers of interferon gamma (IFN-γ), is highly relevant for predicting the therapeutic response to anti-PD-1/PD-L1 therapy [8, 9].

It is becoming increasingly clear that V600E mutation possessed multiple malignancy properties [10] and poor response to MAPK inhibitors [11], and also supported the formation of suppressive TIME [12], and thus correlated to poor prognosis in CRC. BRAF V600E mutation was positively correlated with the expression of TIM3 in both CRC cells and tumor-infiltrating lymphocytes (TILs) [13] as well as the expression of PD-L1 in tumor cells [14], suggesting that it might act as an indicator of ICI treatment efficacy in mismatch repair-deficiency (dMMR) CRC [15]. Furthermore, V600E mutation also increased the infiltration of M2 macrophages via promoting microvessels and microlymphatic vessels formation [12]. However, the immuno-modulatory and biological characteristics of non-V600 BRAF mutations remained unknown in CRC.

In this study, we established three mutant MC38 cell lines possessed V600E (class I), G469V (class II) and D594A (class III) mutation, respectively. We showed that D594A mutant cells were less aggressive and displayed increased expression of immuno-modulatory molecules. Moreover, D594A mutation enhanced the secretion of CXCL9 and CXCL10 via ATF3-THBS1 axis, which was associated with CD8^+^ T cells recruitment and anti-PD-L1 sensitivity. Our work expands the understanding of the biological role and immune related signatures of BRAF D594A mutation in CRC and provides important insights in designing effective immunotherapeutic strategies in clinic.

## Materials and methods

### Establishment of BRAF mutant cell lines

Endogenous *BRAF* gene in MC38 cells was knockout by lenti-CRISPR Cas9 technique. The BRAF WT and mutant plasmids were purchased from Shanghai Jikai Gene Chemical Technology Co., LTD. Briefly, the WT BRAF (NM_004333) gene sequence was synthesized by whole gene synthesis technique. Sequences of V600E (T1799A), G469V (G1406T) and D594A (A1781C) were obtained by point mutation technique. The recombinant plasmids were transfected into BRAF knockout MC38 cell lines respectively according to the instructions (Cat Nr. 101000046, Polyplus, France). Neomycin was added into medium to obtain positive clones (Cat Nr. E859-5G, Amresco).

### Cell culture

MC38 cell line was purchased from ATCC (American Type Culture Collection). The cell line was verified by Short Tandem Repeat (STR) profiling (Shanghai Biowing Applied Biotechnology Company, China). Mycoplasma were detected routinely. MC38 cells were cultured in DMEM (Gibco, USA) medium contained 10% fetal bovine serum (FBS) (Gibco, USA). The medium also supplemented with 100 U/mL penicillin (Cat Nr. SV30010, Hyclone, USA) and 100 μg/mL streptomycin (Cat Nr. SV30010, Hyclone, USA). Cells were maintained in a humidified atmosphere at 37 °C with 5% CO_2_.

### Quantitative reverse transcription polymerase chain reaction (qRT-PCR)

Cells were digested with trypsin (Cat Nr. 03-050-1A, BI, Israel) and washed using PBS. RNA was extracted with TRIzol reagent (Cat Nr. R401-01-AA, Vazyme, China), and reversed transcription into cDNA according to manufacturer's instructions (Cat Nr. RR036A, TaKaRa, Japan). Primers were designed using Primer-BLAST (PubMed) and synthesized from Genewiz Corporation (China). qRT-PCR reactions were performed in triplicate (LightCycler® 480 System, Roche, USA). The expression was normalized using β-actin (5′-GCTACGAGCTGCCTGACGG-3′ for forward primer, and 5′-TGTTGGCGTACAGGTCTTTGC-3′ for reverse primer). The relative expression of genes was calculated by 2^−△Ct^.

### Western blotting analysis

The protein extraction, concentration measurement and imaging were performed referring to the method described before [16]. Rabbit monoclonal anti-mouse BRAF, THBS1, p-ERK, p-MEK, ERK, MEK and Cyclin D1 (Cat Nr. 14814, 37879, 4370, 9154, 4695, 4694 and 2978, Cell Signaling Technology, Germany), rabbit monoclonal anti-rabbit β-actin (Cat Nr. AC026, ABclonal, USA) were used as primary antibodies in the study.

### Colony-forming assay

Exponential growth phase of BRAF WT and mutant MC38 cells were collected. 1,000 cells were seeded in 35 mm dish. The culture medium was changed every 3–4 days. After 10–12 days, clones were washed by PBS gently and fixed using 4% paraformaldehyde for 15 min at room temperature. After that, clones were washed again and stained by 0.1% crystal violet reagent for 20 min. The dishes were washed gently with PBS until the background was clean. Inverted microscope was used to count and photograph.

### Cell viability assay

Cell viability was evaluated by CCK-8 assay (Cat Nr. K1018, APExBio, USA). Cells (2000 cells/well) were seeded into 96-well plate and incubated at 37 °C for 24, 48 and 72 h, respectively. Four replicates were performed. 10 µL CCK-8 reagent was added to each well. After incubation at 37 °C for 2 h, the OD values at 450 nm of each well was measured. The data was analyzed by GraphPad Prism 8.0.

### Cell apoptosis assay

Cells (300,000 cells/well) were seeded into 6-well plate and cultured for 24 h. The trifluoromethoxy carbonylcyanide phenylhydrazone (FCCP) reagent (Cat Nr. S8276, Selleck, USA) was added to induce cell apoptosis for 24 h. All the cells were collected and stained by the APC-Annexin V Binding Apoptosis assay kit (Cat. Nr. 22837; AAT Bioquest, USA) according to the manufacturer's protocol. The proportion of apoptotic cells was analyzed by flow cytometry.

### Cell migration and invasion assays

Transwell chamber (Cat Nr. 3422, Corning, USA) was used in the migration and invasion assay. Before the invasion assay, 100 µL diluted matrigel was added into the upper chamber. Cells were resuspended in DMEM medium (without FBS) at the concentration of 100,000 cells/100 µL or 300,000 cells/100 µL and added to the upper chamber. DMEM medium (600 µL) supplemented with 15% FBS was added in the lower chamber of each well. After incubation for 24 h, cells on the upper chamber were fixed using 4% paraformaldehyde for 15 min. The upper chamber was washed twice with PBS, and stained with 0.1% crystal violet for 20 min. Cells on the upper surface of the filter were wiped by cotton swabs. Cells passing through the transwell membrane were observed and photographed under an inverted microscope.

### Detection of the sensitivity of BRAF mutant and WT cells to MAPK inhibitors

Tumor cells in exponential growth stage were collected. 4,000 cells were seeded into 96-well plate and incubated overnight. On the second day, Sorafenib, Trametinib and SCH772984 (Cat Nr. S7397, S2673 and S7101, Selleck, USA) were serially diluted to the concentrations as 100 µM, 80 µM, 60 µM, 40 µM, 20 µM, respectively, and were added into the wells of each group. Three replicates were performed. The cells were treated with the inhibitors for 24 h, 48 h and 72 h, respectively. The OD values at 450 nm were obtained using CCK-8 method. IC50 values were calculated and the sensitivity of different mutant cell models to MAPK inhibitors were analyzed.

### EGF stimulation experiment

Cells (300,000 cells/well) were incubated in 6-well plate overnight. The culture medium was discarded. The plate was washed gently by pre-warmed PBS. The cells were starved by culturing in FBS-free DMEM medium. After 36 h, the medium in the wells were replaced by 2 mL DMEM medium containing 100 ng/mL EGF (Cat Nr. C029, Novoprotein, China) for 15 min at 37 °C. The whole protein was extracted immediately using RIPA. The control group was avoided contacting with FBS throughout the operation.

### ELISA test for CXCL9 and CXCL10

Cells (300,000 cells/well) were incubated in 6-well plate. 20 ng/mL IFN-γ was added in the culture medium on the second day and treated for 48 h. The supernatant of tumor cells was collected and centrifuged at 4˚C, 4,000 rpm for 10 min to discard the cell debris. The concentrations of CXCL9 (Cat Nr. EK5306, Signalway Antibody, USA) and CXCL10 (Cat Nr. abs520013, Absin, China) were detected using ELISA method follow the manufacturer's instructions. The OD values at 450 nm and 570 nm were detected. The standard curve was drawn and the concentrations of CXCL9 and CXCL10 were calculated.

### Detection of the expression of PD-L1 and MHC Class I in tumor cells by flow cytometry

Tumor cells (300,000 cells/well) were seeded in 6-well plate and incubated overnight. The culture medium was replaced by fresh complete medium containing 20 ng/mL IFN-γ. The tumor cells were harvested 48 h later and washed by PBS. Anti-mouse-PD-L1 antibody (Cat Nr. 17–5982-82, Invitrogen, USA) and anti-mouse-MHC Class I antibody (Cat Nr. 141603, Biolegend, USA) were diluted using FACS buffer (PBS containing 10% FBS) at the ratio of 1:200. The cells were resuspended by the dye solution and stained for 25 min on ice. After washing with FACS buffer, cells were resuspended by FACS buffer and filtered by nylon membrane. The single cell suspension was analyzed by BD LSRFortessa™ Cell Analyzer (BD Biosciences, USA). The data was processed using flowjo Vx 10.0 software.

### Detection of the function of T cells co-cultured with tumor supernatant by flow cytometry

The supernatant of tumor cells was collected as above. Lymph nodes from OT-1 mice were grinded, and T cells were collected to be activated with IL-2 and N4-peptide for three days. Live T cells were collected by density gradient centrifugation. 500,000 T cells were incubated in medium containing equal volume of T cells culture medium and tumor supernatant. Three replicates were performed. After co-culture for 48 h, the expression of functional markers of CD8^+^ T cells, TNF-α, IFN-γ, PD-L1 and TIM3 were detected by flow cytometry.

PD-1 (Cat Nr. 46–9981-82, Invitrogen, USA) and TIM3 (Cat Nr. 25–5870-82, Invitrogen, USA) were detected as above. For the detection of secretory TNF-α and IFN-γ, T cells were firstly incubated in culture medium containing PMA (5 ng/mL), Ionomycin (500 ng/mL), Brefeldin A (1: 1,000) and Monensin (1: 1,000) for 4 h at 37 °C. T cells were collected and resuspended by FACS buffer. Fixable viability dye efluor 506 (Cat Nr. 65–0866-18, Invitrogen, USA) was added for staining for 25 min on ice. Then T cells were washed and resuspended in 1 mL freshly prepared Fix/Perm solution (BD Biosciences) for 20 min at 4 °C. After being washed with Perm/Wash buffer (BD Biosciences), T cells were stained with anti-TNF-α (Cat Nr. 506304, Biolegend, USA) and anti-IFN-γ (Cat Nr. 505810, Biolegend, USA) for 25 min, washed and filtered. All samples run on a flow cytometer and analyzed using flowjo V× 10.0 software.

### Establishment of the animal models and anti-PD-L1 treatment

C57BL/6 and BALB/C Nude female mice were purchased from Beijing Vital River Laboratory Animal Technology Co., Ltd. and Cavens Biogle Model Animal Research Co.,Ltd. (China), respectively. BRAF mutant and WT cell lines at exponential growth stage were prepared and resuspended by PBS. 1,000,000 cells were injected subcutaneously on the right flank of the mice. Tumor volumes were measured every 2–3 days. The long diameter (L) and short diameter (W) of the tumors were measured, and tumor volume was calculated as: volume = (LW^2^)/2. All animal procedures were conducted in accordance with the guidelines of the Animal Care and Welfare Committee of Nanjing medical university.

In the ICIs sensitivity experiment, tumor cells were injected subcutaneously as above. The mice were randomly divided into two groups on day 9. 200 mg Atezolizumab was injected intraperitoneally three times per week to each mouse of the experimental group. The tumor growth curve was drawn according to tumor volumes. The tumors were weighed when the mice were sacrificed.

### Analysis of TILs by flow cytometry

Once the mice were sacrificed, the tumors were dissected and grinded. Isolated cells were resuspended in FACS buffer and incubated with Fc-block for 20 min first. Monoclonal antibodies of CD45, CD3, CD4, CD8, NK1.1, Ly6G, Ly6C, CD25, CD103, PD-1 and TIM3 were added for surface staining. The procedure of flow cytometry was as mentioned above.

### Immunohistochemistry (IHC) staining for CD8α

Tumor tissues were harvested from tumor-bearing mice and stored in formalin at 4 °C. The tissue was embedded in paraffin and prepared into serial slices. After dewaxing and antigen retrieval, the background was blocked. The slices were then incubated with primary antibody specific to CD8α (Cat Nr. 98941, Cell Signaling Technology, Germany) in a wet box at 4 °C overnight. The slices were washed with PBS for 4 times and incubated with secondary antibody at 37 °C for 15 min. The color reaction was performed using DAB Substrate kit (Cat Nr. DA1010, Solarbio, China). The slices were observed and photographed under an inverted microscope. The positive cells were counted and analyzed by GraphPad Prism 8.0.

### RNA sequencing

BRAF WT and D594A mutant MC38 cells were harvested at exponential growth stage. Total RNA was isolated using Trizol reagent. RNA quality and integrity were confirmed via an Agilent Bioanalyzer 2100. Then, mRNA libraries were established and sequenced at Genewiz Corporation (China).

### Establishment of THBS1-overexpressing cells

THBS1-overexpressing plasmid (pMSCV-THBS1) was constructed based on pMSCV-PIG (Cat Nr. 18751, Addgene) by homologous recombination technique (Cat Nr. C112, Vazyme, China). Briefly, the sequence of THBS1 was obtained by cloning the MC38 cDNA template using the following primers; forward primer, 5′-gccggaattagatctctcgagATGGGGCTGGCCTGGGGACTA-′3 and reverse primer, 5′- gtagaattcgttaacctcgagTTAATGGTGATGGTGATGATGGGGATCTCTACATTCGTATTTCAGGT-′3, and recombined to linearized pMSCV-PIG at XhoI restriction site. THBS1-overexpressing (hTHBS1) and control (hctrl) cell lines were established by transfecting either pMSCV-THBS1 plasmid or pMSCV-PIG into MC38 cells using jetPRIME reagents (Cat Nr. 101000046, Polyplus, France). qRT-PCR was used to determine *THBS1* mRNA expression using the following primers: forward primer, 5′- GGGGAGATAACGGTGTGTTTG-′3, and reverse primer, 5′- CGGGGATCAGGTTGGCATT-3′. Western blot was used to detect the THBS1 protein expression in cells.

### Establishment of THBS1 knockdown cells

The shRNA sequences targeting THBS1 were as following; sense: CCGGTGAAACCGATTTCC GACAATTCTCGAGAATTGTCGGAAATCGGTTTCATTTTTGA; anti-sense: ATTCAAAAATGAAACCGATTTCCGACAATTCTCGAGAATTGTCGGAAATCGGTTTCA. THBS1-knockdown plasmid (pLKO.1-shTHBS1) was constructed based on pLKO.1 (Cat Nr. 8453, Addgene) by homologous recombination technique. THBS1-knockdown (siTHBS1) and control (sictrl) cell lines were established by transfecting either pLKO.1-shTHBS1 or pLKO.1 plasmid into D594A mutant MC38 cells using jetPRIME reagents (Cat Nr. 101000046, Polyplus, France). Cells were maintained in a culture medium containing 400 μg/mL neomycin (Cat Nr. E859-5G, Amresco) for two weeks. qRT-PCR and western blot were used to determine the mRNA and protein levels of THBS1.

### T cells migration assay

Transwell chamber (Cat Nr. 3422, Corning, USA) was used in the T cell migration assay. CD8^+^ T cells were resuspended in DMEM medium at the concentration of 500,000 cells/120 µL and added into the upper chamber. Culture supernatant (600 µL) from tumor cells was added to the lower chamber of each well. 0.1 µg/mL CXCL9 and CXCL10 neutralizing antibody were used. After incubation for 3 h, cells that migrated through the filter was counted and photographed under an inverted microscope. Three replicates were performed.

### Mice models treated by CXCL9 or CXCL10 neutralizing antibody

hTHBS1 and hctrl cells were injected subcutaneously into C57BL/6 mice respectively. The mice injected hTHBS1 cells were divided into three groups randomly. Two experimental groups were given 5 µg/100 µL CXCL9 and CXCL10 neutralizing antibody by injecting intraperitoneally, respectively. The remaining group was given 100 µL PBS. The injection was performed on day 8, 11 and 14 after tumor transplantation. The tumor growth curve was drawn and the tumors were weighed. TILs were analyzed by flow cytometry.

### Statistics

All experiments were repeated at least three times. Data were expressed as the mean ± standard deviation. The differences between two groups were analyzed by non-parametric *t*-test. *P* < 0.05 was considered to indicate a statistically difference. All statistical analyses were two-sided and performed by GraphPad Prism 8.0. Figures were drawn using GraphPad Prism 8.0.

## Results

### D594A mutation confers lower aggressiveness on MC38 cells

**Fig. 1:**
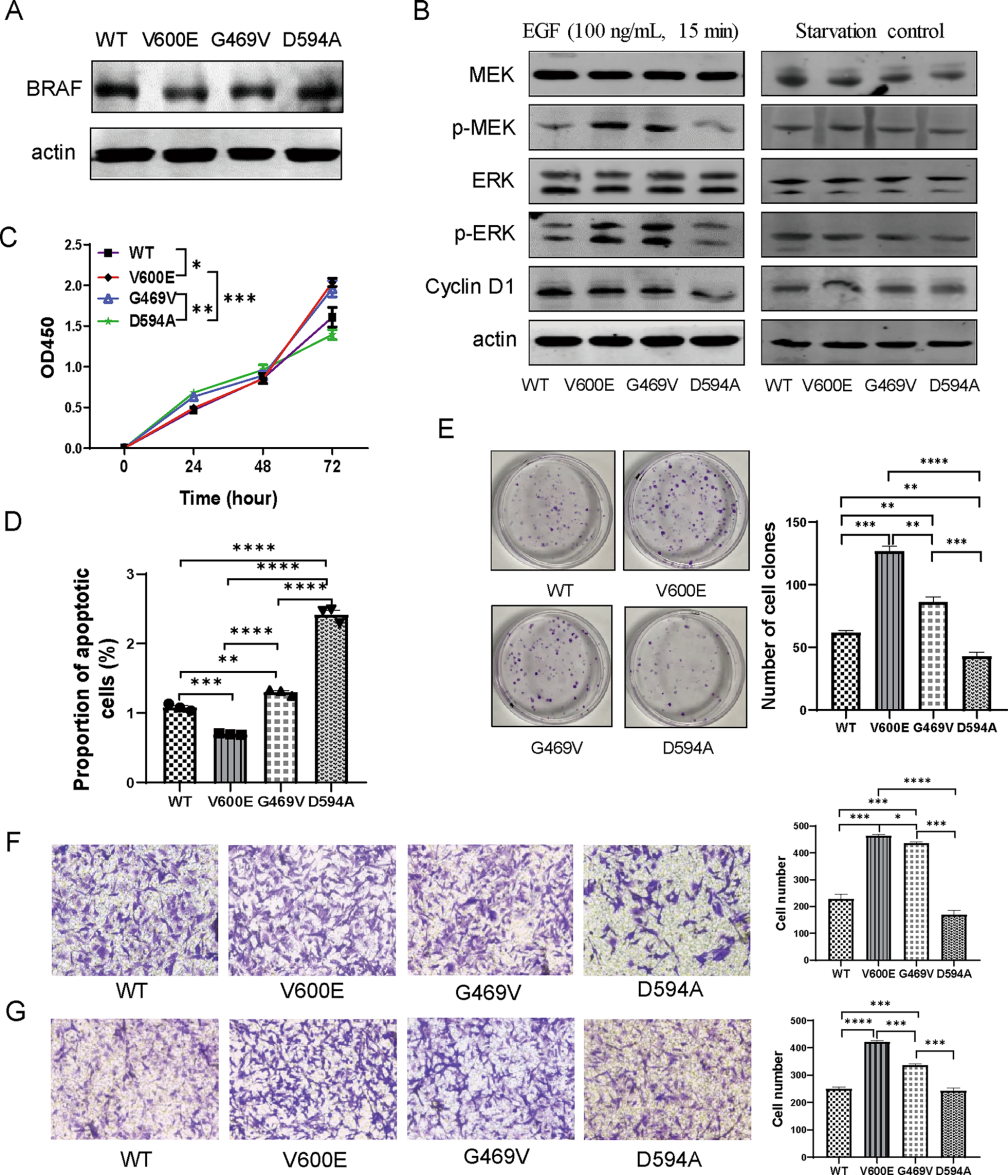
D594A mutation confers lower aggressiveness on MC38 cells. **A** Immunoblotting of BRAF level in WT and mutant MC38 cells. Actin serves as an internal reference. **B** Representative immunoblotting of MEK, pMEK, ERK1/2, pERK1/2 and Cyclin D1 in the starvation group (right) and stimulation group (left). Cells were exposed to EGF (100 ng/mL) for 15 min after starvation for 36 h in stimulation group. **C** Proliferation ability of BRAF WT and mutant MC38 cells at 24 h, 48 h and 72 h detected by CCK-8 assay. **D** Detection of apoptosis by annexin V/PI staining after induction of FCCP for 36 h. **E** Growth ability of BRAF WT and mutant MC38 cells analyzed by clone formation assay. **F**, **G** Migration and invasion abilities of BRAF WT and mutant MC38 cells analyzed by transwell assays, respectively. Three independent replicates were performed for above experiments. *P < 0.05; **P < 0.01; ***P < 0.001; ****P < 0.0001

To investigate the potential biological impacts of distinct BRAF mutations on CRC cells, BRAF V600E (class I), G469V (class II), D594A (class III) mutant and WT MC38 stable cell lines were established by virus infection. As shown by western blot, no significant differences in protein expression of BRAF were observed among the four established cells (Fig. 1A). The mutation sites were verified by sequencing (Additional file 1: Fig. S1A). To further explore the potential impacts of distinct BRAF mutations on MAPK pathway activation, BRAF mutant and WT cells were exposed to 100 ng/mL EGF for 15 min after serum deprivation. As expected, V600E mutation induced the strongest phosphorylation of ERK signaling cascade, whereas G469V and D594A mutations showed modest activation. The activation status of MAPK signaling was further validated with the expression pattern of downstream molecule Cyclin D1 (Fig. 1B).

We further examined the impacts of different BRAF mutations on cell growth. Compared to WT cells, cells harboring V600E mutation exhibited stronger growth ability, but the growth rate was not altered with D594A mutation, suggesting that D594A mutation may have little effects on tumor proliferation or progression (Fig. 1C). Next, we examined the impacts of different BRAF mutations on MC38 cell survival. D594A cells were more fragile and had higher proportion of apoptotic population in response to FCCP treatment, whereas V600E reduced Annexin V positive population compared with WT (Fig. 1D). As expected, colony formation showed that D594A mutation displayed a collapse of expansion capacity, similar with its proliferative ability as compared with WT, while V600E and G469V could enhance cell expansion obviously (Fig. 1E). Migration and invasion abilities are also tumor malignant features. Transwell assays revealed that V600E and G469V could enhance the abilities of cell migration and invasion, while D594A reduced cell migration and did not alter cell invasion compared with WT (Fig. 1F, G). We further examined the impacts of different BRAF mutations on response sensitivity to MAPK inhibitors, D594A was the most sensitive to BRAFi and MEKi, while V600E was relatively sensitive to ERKi (Additional file 1: Fig. S1C).

In short, CRC cells with distinct classes of BRAF mutation present biological behaviors with different malignancy degree. Overall, the D594A mutation was demonstrated to display relatively weaker expansion and metastasis capacity than V600E and G469V mutations.

### BRAF mutation modulates the expressions of PD-L1 and MHC class I, which were the highest in D594A

**Fig. 2:**
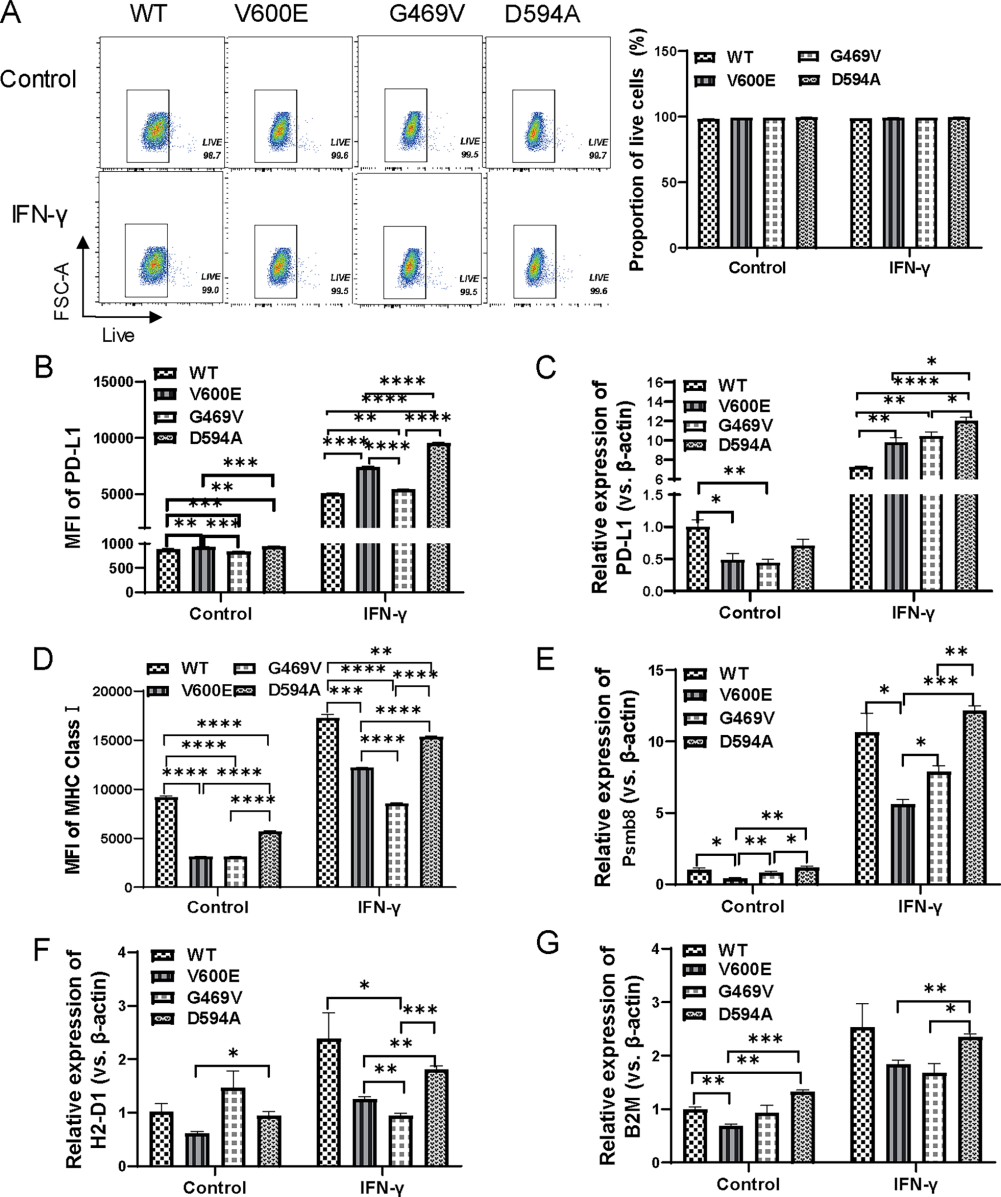
BRAF mutation modulates the expression of immune regulatory molecules. **A** The effect of IFN-γ treatment on the survival of tumor cells analyzed by flow cytometry. **B**, **C** The basal and induced expressions of PD-L1 in BRAF WT and mutant MC38 cells detected by flow cytometry and qRT-PCR. **D** The basal and induced expressions of MHC class I in BRAF WT and mutant cells detected by flow cytometry. **E**–**G** The basal and induced expressions of *Psmb8*, *H2-D1* and *B2M* in BRAF WT and mutant cells detected by qRT-PCR. Cells were treated by 20 ng/mL IFN-γ for 48 h. Three independent replicates were performed for above experiments. *P < 0.05; **P < 0.01; ***P < 0.001; ****P < 0.0001

Tumor cells in the TIME would be exposed to IFN-γ secreted by NK cells or activated cytotoxic T cells. Of note, IFN-γ stimulates the expressions of immune regulatory molecules in tumor cells, like major histocompatibility complex (MHC) class I and II, PD-L1 and PD-L2. MHC class II and PD-L2 were not expressed in MC38 cells in our previous and other’s studies [17]. We thus detected the basal and inducible expressions of PD-L1 and MHC class I in these tumor cells with distinct BRAF mutations by flow cytometry and qRT-PCR. 20 ng/mL IFN-γ treatment didn’t cause damage to cancer cells (Fig. 2A). Little differences were seen regarding the basal expression of PD-L1 among the four classified cells. Interestingly, D594A significantly upregulated the surface expression of PD-L1 compared with V600E and G469V upon IFN-γ stimulation (Fig. 2B). Consistently, the mRNA expression of *PD-L1* was also the highest in D594A among the four classified cells upon IFN-γ treatment (Fig. 2C).

BRAF mutation downregulated the expression of MHC class I on both protein and mRNA levels. Notably, cells harboring D594A mutation expressed higher MHC class I than V600E and G469V with or without IFN-γ induction (Fig. 2D). IFN-γ treatment significantly increased the mRNA levels of *Psmb8* (Fig. 2E), *H2-D1* (Fig. 2F) and *B2M* (Fig. 2G), three important molecules in the process of antigen presentation, all of which were the highest in D594A cells. Thus, D594A mutation not only upregulated the level of immunosuppressive PD-L1, but also promoted the expression of antigen presentation molecules.

### D594A mutation altered the landscape of the tumor microenvironment

**Fig. 3:**
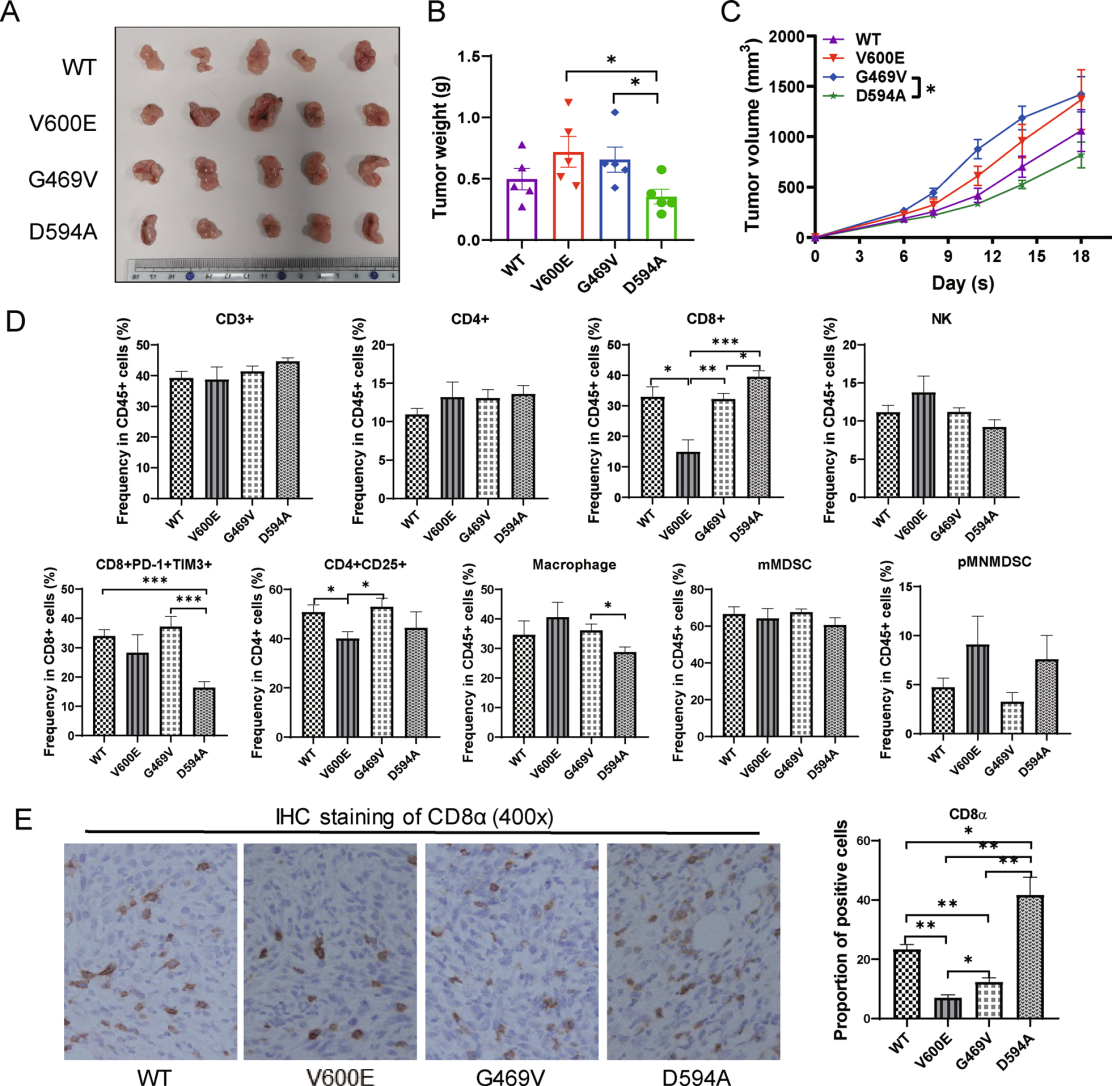
BRAF mutation influences the panel of TILs in transplanted tumor. BRAF WT and mutant MC38 cells were inoculated subcutaneously in homologous C57BL/6J mice (n = 5 mice per group). The tumor sizes, weights and growth curves of the four groups were shown as (**A**–**C**), respectively. **D** Analysis of the population of TILs by flow cytometry, including CD3, CD4, CD8, NK cells and Macrophage. **E** Representative immunohistochemical staining for CD8α in paraffin section of transplanted tumors (magnification 400 ×). *P < 0.05; **P < 0.01; ***P < 0.001

To determine whether BRAF mutation remodel the tumor microenvironment in respect of immune system, immuno-compromised mice were transplanted subcutaneously with the four classified cancer cells. The tumor volume and weight in G469V group were significantly bigger than those in WT group. No significant differences were observed regarding tumor volume and weight among the three mutant groups (Additional file 2: Fig. S2A-C). However, V600E and G469V enhanced the tumor growth in immunocompetent C57BL/6 mice compared with WT and D594A, suggesting that distinct BRAF mutations may result in alterations of key components of the immune system (Fig. 3A–C).

To assess the composition and functionality of immune cells within the TIME with distinct BRAF mutation, diverse subsets of effector or immunosuppressive populations were analyzed by flow cytometry. The proportion of CD8^+^ T cells was significantly increased in D594A compared to that in V600E, G469V and WT, which was also confirmed by IHC (Fig. 3E). However, the frequency of natural killer (NK) cells was relatively reduced in D594A than in the other three groups. Strikingly, the percentage of terminally exhausted CD8^+^PD-1^+^TIM3^+^ population in D594A was the lowest among the four groups, indicating improved CD8^+^ T cell effector functions in D594A TIME. The proportions of CD3^+^, CD4^+^ T cells, pMN/MDSC and mMDSC populations were not altered among the four transplanted tumors (Fig. 3D).

Therefore, distinct BRAF mutation may reprogram the composition of diverse immune subsets of the TIME respectively compared with WT. In particular, the frequency of tumor infiltrated CD8^+^ T cells was increased whereas the expression of immunoinhibitory molecules was blunted in tumors with D594A mutation.

### *D594A mutation enhances CD8*^+^*T cell effector function *in vitro

**Fig. 4:**
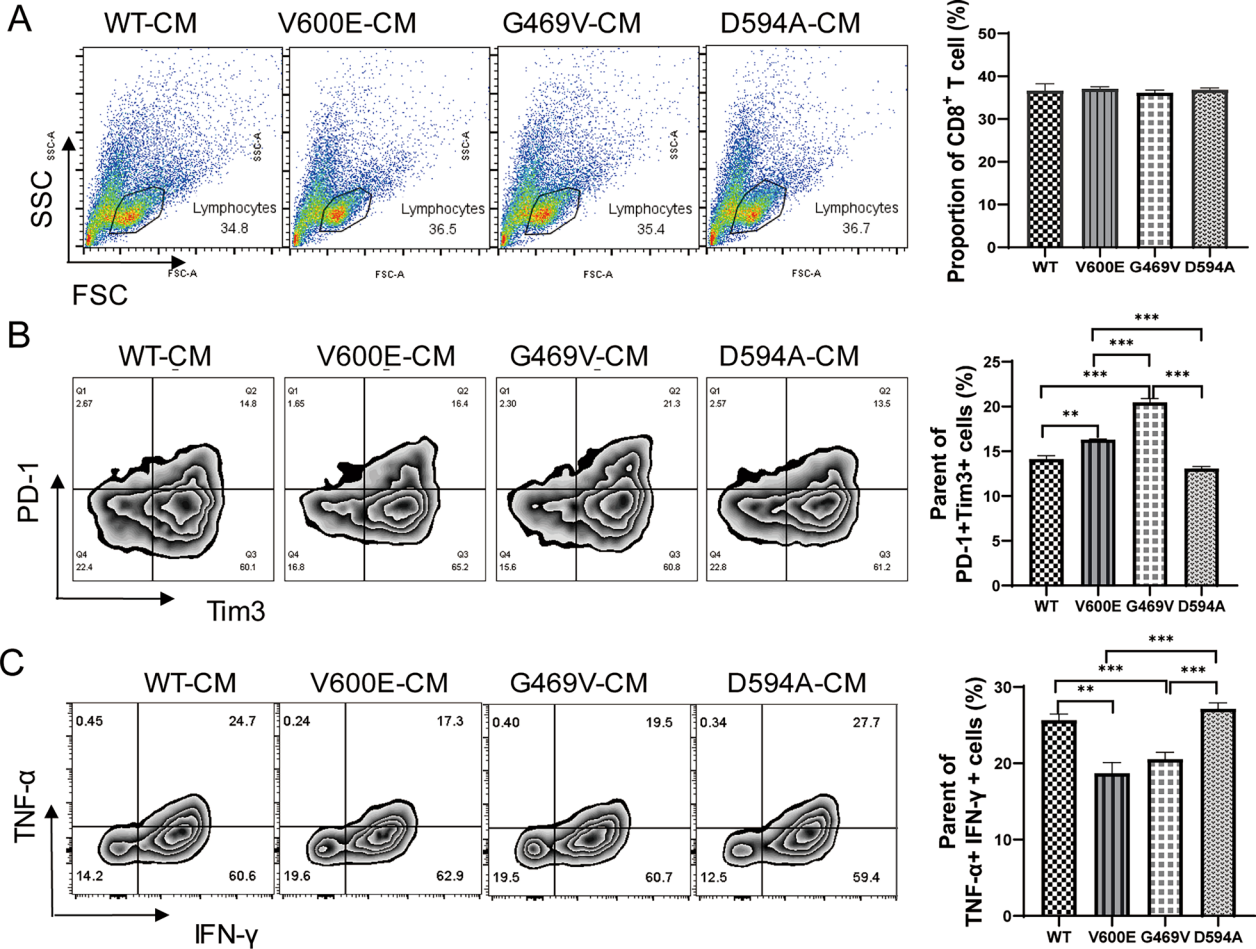
D594A mutation enhances CD8^+^ T cell effector function in vitro. **A** Analysis of the survival of CD8^+^ T cells by flow cytometry. **B**, **C** Analysis of the population expressing PD-1, TIM3, TNF-α and IFN-γ in CD8^+^ T cells by flow cytometry. CD8^+^ T cells were co-cultured with different tumor supernatants for 48 h. Three independent replicates were performed for above experiments. **P < 0.01; ***P < 0.001

Cytotoxic CD8^+^ T cells play critical roles to fight against tumor. To further explore the effects of BRAF mutation on CD8^+^ T cell effector function, we co-cultured CD8^+^ T cells with supernatant derived from different BRAF mutant cells in vitro. There is no difference among these mutations in terms of the impact on viability of CD8^+^ T cells (Fig. 4A). Surprisingly, D594A-derived supernatant dramatically decreased TIM3 and PD-1 expression levels on CD8^+^ T cells, which is accompanied with enhanced TNF-α and IFN-γ production as compared with V600E and G469V cells (Fig. 4B, C).

### D594A mutation leads to transcriptional reprograming of cell chemotaxis

**Fig. 5:**
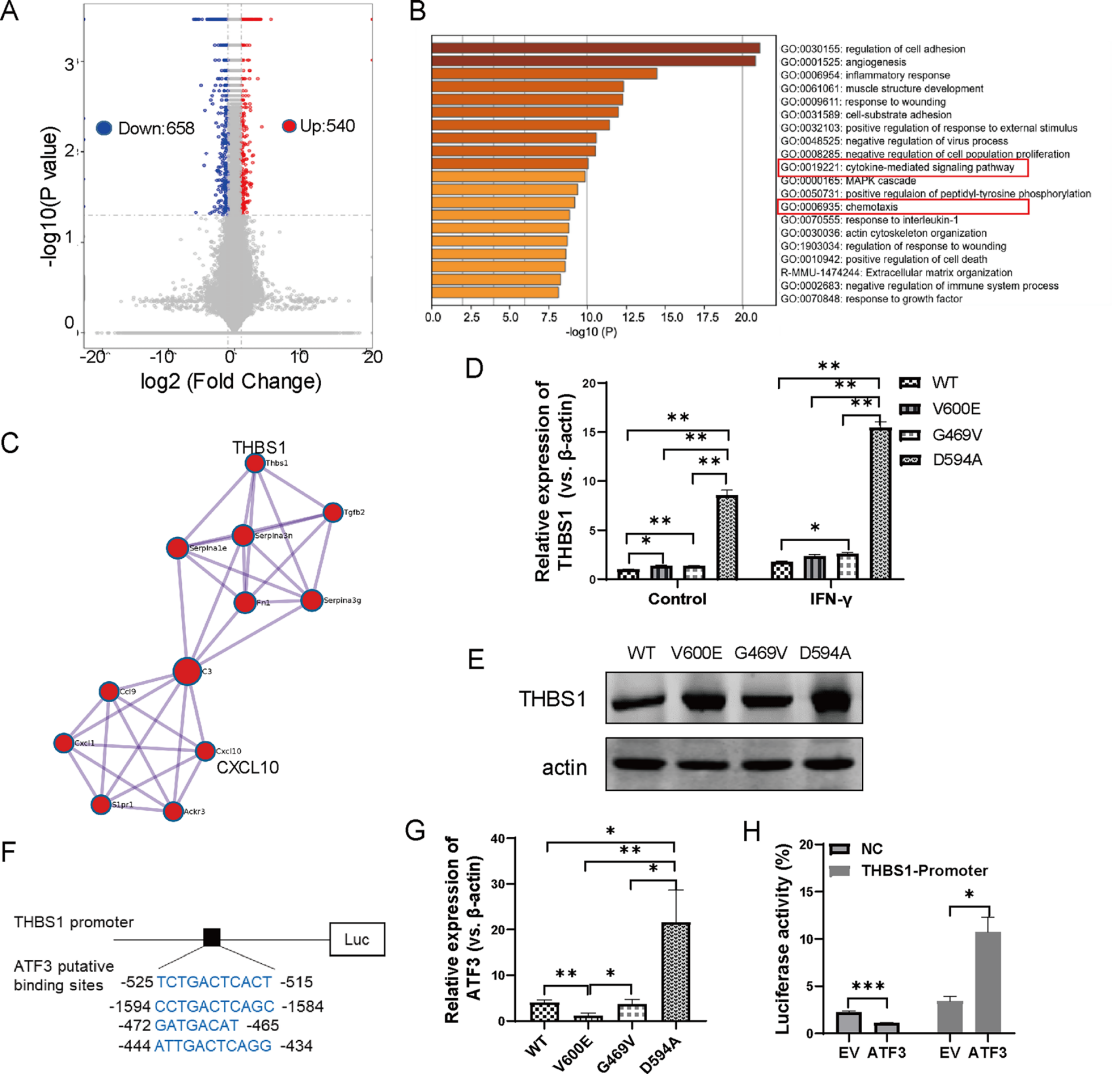
D594A mutation reprograms transcriptome involved in cell chemotaxis and upregulates THBS1 expression via ATF3. **A** Analysis of differentially expressed genes (DEGs) between D594A and WT cells by RNA-seq. **B** Functional enrichment of DEGs by online Metascape analysis. **C** The potential relationship between THBS1 and CXCL10 revealed by bioinformation prediction. **D** The basal and induced expressions of *THBS1* in BRAF WT and mutant cells detected by qRT-PCR. Cells were treated by 20 ng/mL IFN-γ for 48 h. **E** The protein level of THBS1 in BRAF WT and mutant cells detected by western blot. **F** The putative binding sites of ATF3 on THBS1 promoter predicted by jaspar website. **G** The *ATF3* mRNA level in BRAF WT and mutant cells detected by qRT-PCR. **H** The transcriptional regulation of THBS1 by ATF3 examed by dual-luciferase reporter assay. EV, empty vector; NC, negative control. *P < 0.05; **P < 0.01; ***P < 0.001

Based on the aforementioned observations, we found that D594A mutation not only weakened the malignancy of MC38 cells, but also resulted in immune activation in vivo. D594A mutation induced a better tumor regression compared with V600E and G469V. Considering the lower infiltration of NK cells, we speculated that the frequency and function of infiltrated CD8^+^ T cells were the key effectors to control cancer with D594A mutation. To explore the mechanism underlying the recruitment of CD8^+^ T cells, the differential expressed genes (DEGs) between D594A and WT cells were analyzed by RNA sequencing (RNA-seq). Compared to WT, 540 genes were upregulated, and 658 genes downregulated in D594A (Fig. 5A). Cytokine-mediated signaling pathway and chemotaxis were enriched by the online Metascape analysis (Fig. 5B), which are essential in regulating the migration of immune cells [18]. Thus, D594A mutation reprogramed the transcription of DEGs involved in chemotaxis. These in silico findings were further validated by qRT-PCR analysis of representative chemotaxis genes like *THBS1*, *B2M*, *CCL9*, *IFLH1*, *C3*, *CPL3A1*, *HFE* and *IL4RA*. (Additional file 3: Fig. S3).

### *D594A mutation upregulates THBS1 expression *via* ATF3*

Among genes validated above, *THBS1* was predominantly elevated in D594A compared with the other three cell lines. The bioinformatic analysis predicted that THBS1 was associated with CXCL10 (Fig. 5C), which is a key chemokine mediating the recruitment of CD8^+^ T cells. Moreover, the mRNA level of *THBS1* could be further upregulated in response to IFN-γ signaling in D594A (Fig. 5D). The protein expression of THBS1 was also elevated in D594A (Fig. 5E).

*THBS1* gene has transcription regulating sequence (TSRs) in its promoter. To further explore the potential transcriptional factors mediating the upregulation of THBS1, we made a prediction using an online tool (http://jaspar.genereg.net), which showed that *THBS1* can be regulated by numerous transcriptional factors, among which, qRT-PCR verified that ATF3 expressed excessively higher in D594A (Fig. 5F, G). Dual luciferase reporter assay showed that the luciferase activity was drastically boosted only in group co-transfected pGL3-THBS1 promoter and pcDNA 3.1-ATF3 (Fig. 5H). Herein, D594A mutation enhanced *THBS1* expression via reprogramming transcriptional factor *ATF3*.

### THBS1 improves CXCL9/CXCL10 chemokines release in D594A mutant cells

**Fig. 6:**
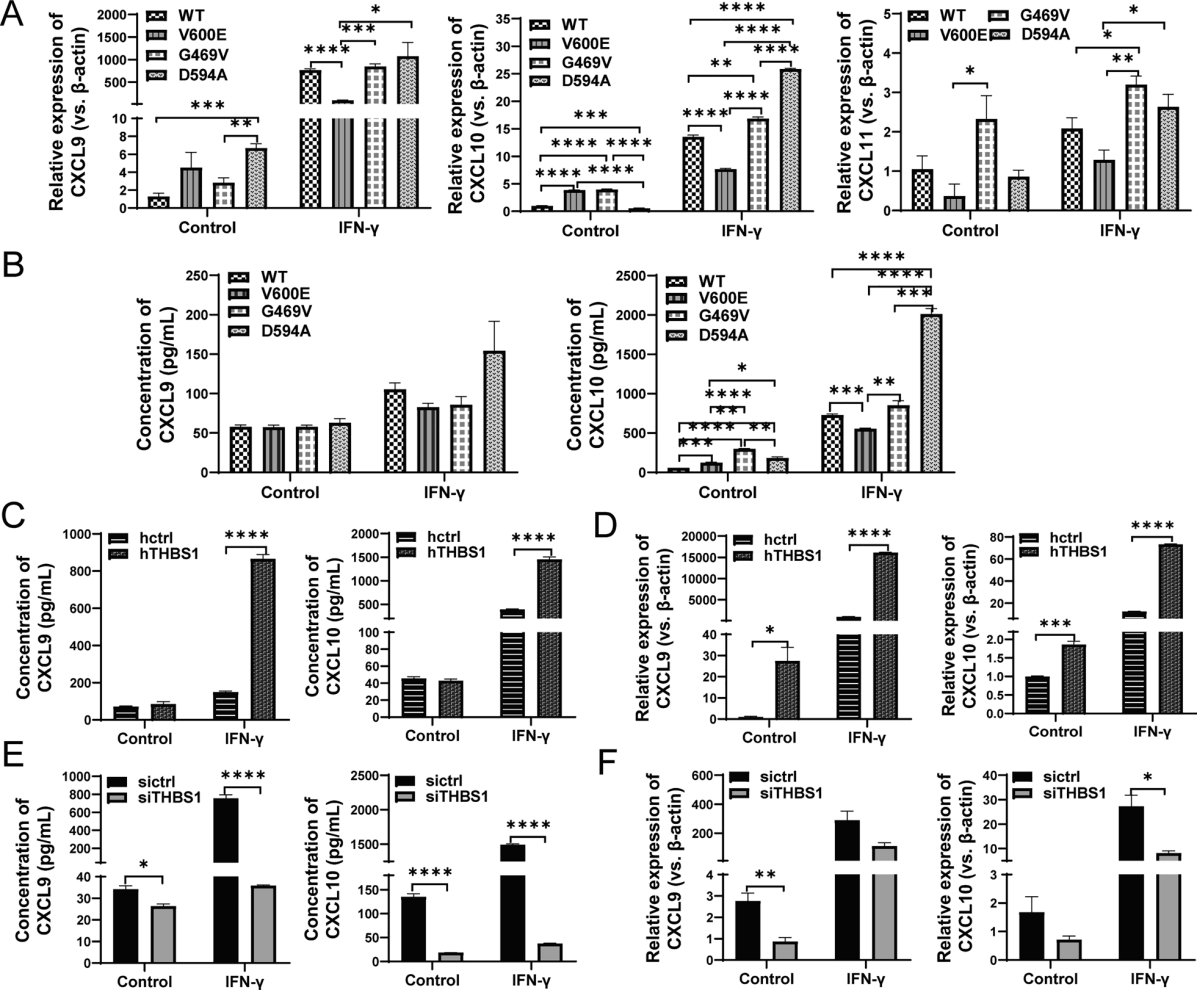
THBS1 improves CXCL9/CXCL10 chemokines release in D594A mutant cells. **A** The mRNA levels of *CXCL9*, *CXCL10* and *CXCL11* in BRAF WT and mutant MC38 cells detected by qRT-PCR. **B** The concentrations of CXCL9 and CXCL10 in the supernatant of BRAF WT and mutant cells analyzed by ELISA. **C** The concentrations of CXCL9 and CXCL10 in the supernatant of hTHBS1 and hctrl cells detected by ELISA. **D** The mRNA expressions of *CXCL9* and *CXCL10* in hTHBS1 and hctrl cells detected by qRT-PCR. **E** The concentrations of CXCL9 and CXCL10 in the supernatant of siTHBS1 and sictrl cells analyzed by ELISA. **F** The mRNA expressions of *CXCL9* and *CXCL10* in siTHBS1 and sictrl cells detected by qRT-PCR. Cells were treated by 20 ng/mL IFN-γ for 48 h. Three independent replicates were performed for above experiments. *P < 0.05; **P < 0.01; ***P < 0.001; ****P < 0.0001

IFN-γ induced chemokines CXCL9, CXCL10 and CXCL11, exert strong chemoattractant effect on C-X-C motif receptor 3 (CXCR3)-expressing CD8^+^ T cells. To this end, we detected *CXCL9*, *CXCL10* and *CXCL11* mRNA levels by qRT-PCR. Of note, the mRNA levels of *CXCL9* and *CXCL10* in D594A cells were significantly higher than those in WT, V600E and G469V cells upon IFN-γ treatment, while *CXCL11* was evidently induced only in G469V cells (Fig. 6A). In addition, the secretion of CXCL9 and CXCL10 in D594A supernatant was also remarkably increased upon IFN-γ treatment by ELISA (Fig. 6B).

To further investigate the role of THBS1 in the secretion of CXCL9/CXCL10, we established THBS1-overexpressing cell line (hTHBS1, Additional file 4: Fig. S4A, B) and THBS1-knockdown cell line (siTHBS1, Additional file 4: Fig. S4C, D), respectively. THBS1 overexpression in MC38 cells significantly enhanced CXCL9 and CXCL10 production and secretion upon IFN-γ treatment compared to hctrl (Fig. 6C, D). Meanwhile, THBS1 knockdown in D594A cells strongly reduced CXCL9 and CXCL10 expression and their secretory capacity compared to sictrl (Fig. 6E, F). Altogether, our data suggest that THBS1 can act as a regulator to promote CXCL9 and CXCL10 expression and release.

### *THBS1-CXCL9/CXCL10 axis promotes the recruitment of CD8*^+^*T cells *in vitro* and *in vivo

**Fig. 7:**
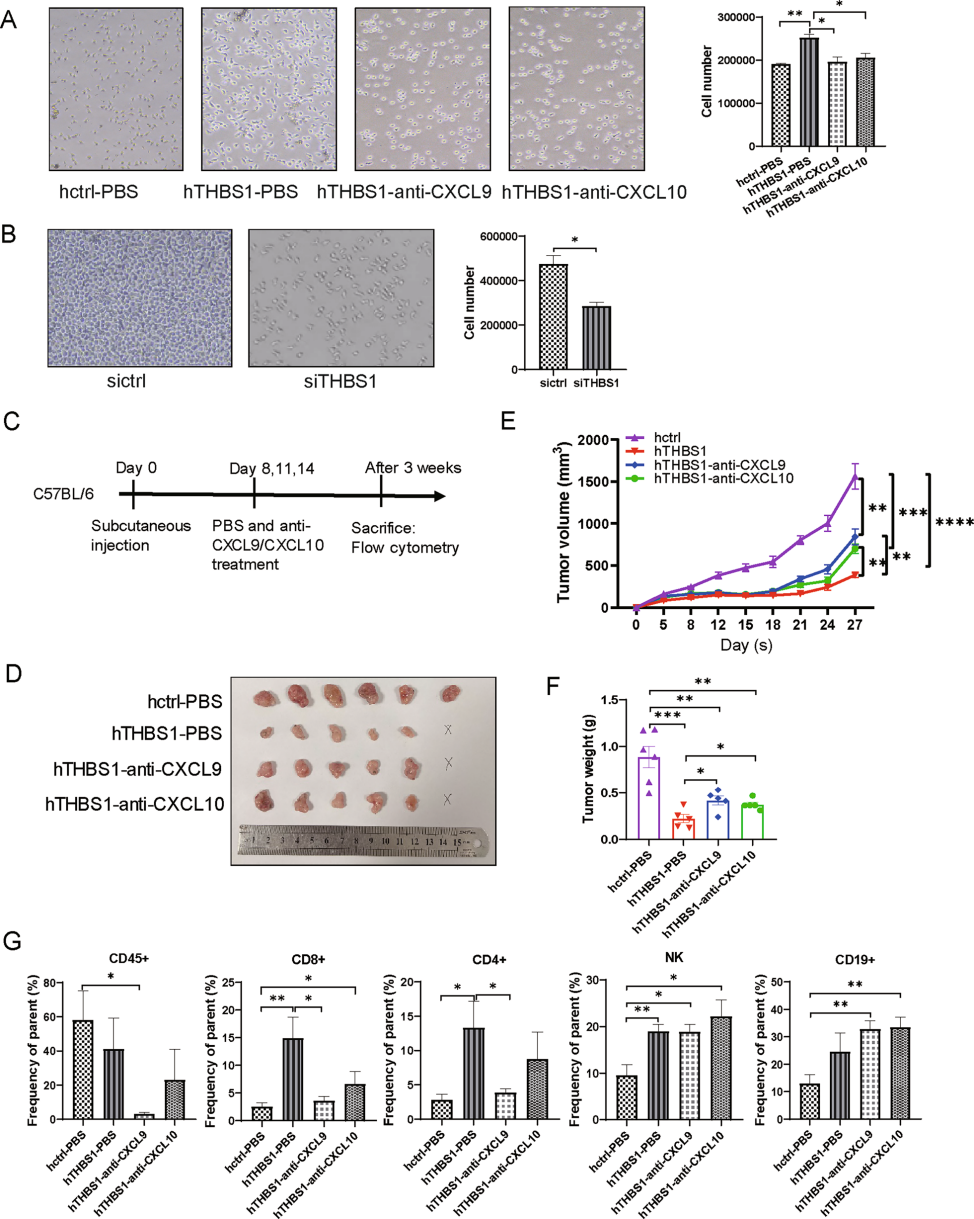
THBS1-CXCL9/CXCL10 axis promotes the recruitment of CD8^+^ T cells. **A** The migration of CD8^+^ T cells coincubated with supernatant from hctrl and hTHBS1 cells, and supernatant handling with CXCL9 or CXCL10 neutralizing antibody. **B** The migration of CD8^+^ T cells coincubated with supernatant from siTHBS1 or sictrl cells. hctrl and hTHBS1 cells were inoculated subcutaneously (n = 6 mice per group). CXCL9/CXCL10 neutralizing antibody or PBS was administrated according to (**C**). The tumor sizes, growth curves and weights of the three groups were shown as (**D**–**F**), respectively. **G** Analysis of the population of TILs by flow cytometry. *P < 0.05; **P < 0.01; ***P < 0.001; ****P < 0.0001

Next, we sought to further explore the role of THBS1-CXCL9/CXCL10 axis in CD8^+^ T cells recruitment. Using an in vitro T cell migration assay, we confirmed that THBS1 overexpression in MC38 cells promoted the migration of CD8^+^ T cells, whereas neutralizing CXCL9 and CXCL10 abolished the promotion (Fig. 7A). Otherwise THBS1 knockdown weakened the migration of CD8^+^ T cells (Fig. 7B). Moreover, we observed that THBS1 overexpression in MC38 cells decreased its tumorigenesis ability in vivo. Neutralizing CXCL9 or CXCL10 partly recovered tumor growth (Fig. 7D–F). Flow cytometry revealed that the frequency of hTHBS1 infiltrated CD8^+^ T cells was abundant in tumor bed. Giving that CXCL9 neutralizing antibody significantly reduced CD8^+^ T cells recruitment, an evident reduction was also observed in CXCL10 neutralizing group while the difference was not significant. The infiltration of CD3^+^CD4^+^ T cells in the four groups were similar as CD8^+^ T cells. Neutralizing antibodies against CXCL9 or CXCL10 didn’t reduce CD19^+^ and NK cells recruitment (Fig. 7G). In this regard, we provide compelling evidences that THBS1-CXCL9/CXCL10 axis plays critical roles in CD8^+^ T cells recruitment and subsequent anti-tumor immunity.

### D594A mutant results in tumor repression upon PD-L1 blockade

**Fig. 8:**
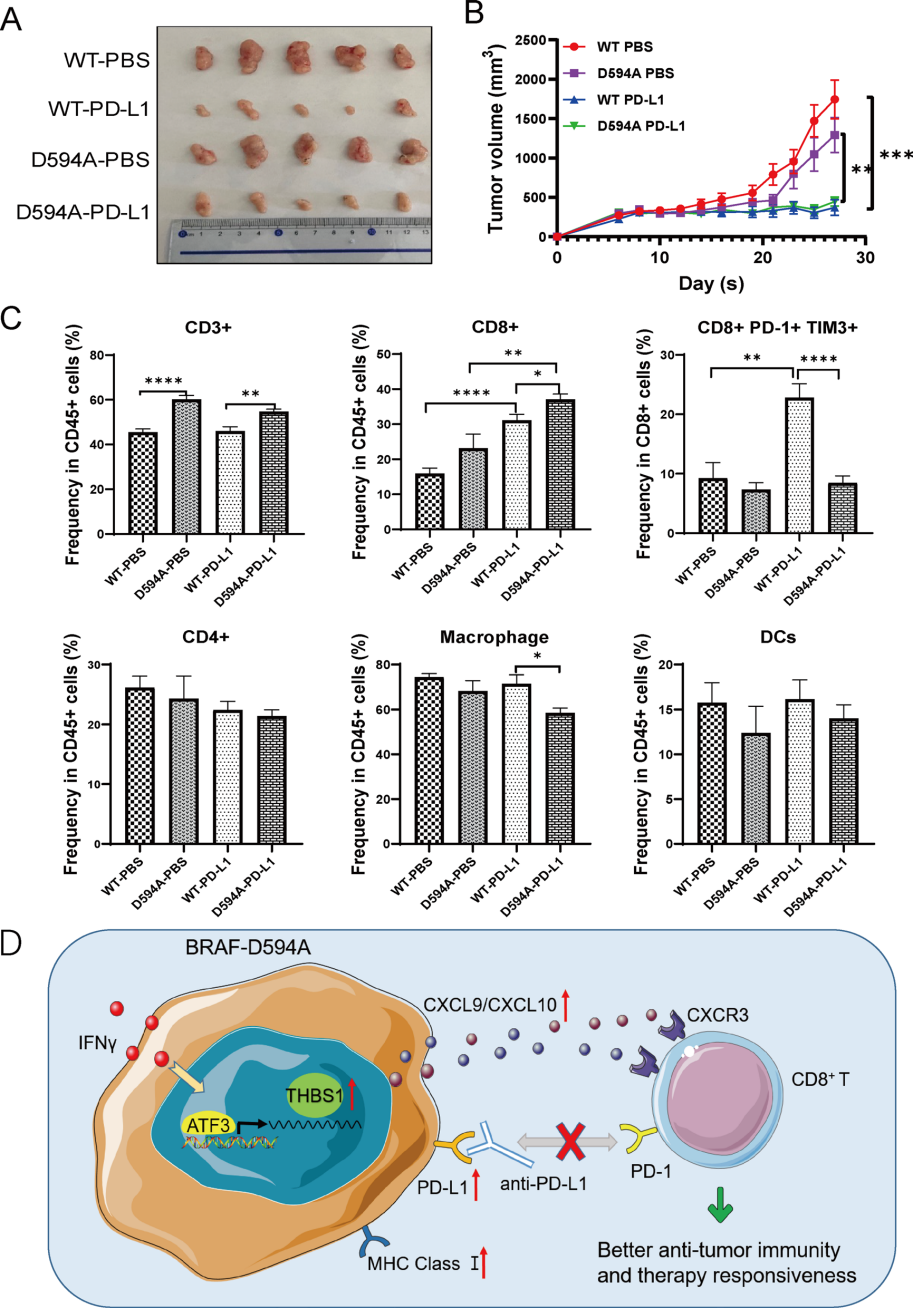
D594A mutant results in tumor repression upon PD-L1 blockade. WT and D594A mutant MC38 cells were inoculated subcutaneously (n = 5 mice per group). Anti-PD-L1 was administrated on day 9 and three times a week. The tumor sizes and growth curves of treated and control groups were shown as (**A**, **B**). **C** Analysis of the population of TILs by flow cytometry, including CD3, CD4, CD8, Macrophage and DC cells. **D** A schematic show that the upregulation of THBS1 promotes the secretion of CXCL9/10 and enhances CD8^+^ T cells recruitment into D594A, thereby enhancing the anti-PD-L1 response, together with the upregulation of MHC class I and PD-L1. *P < 0.05; **P < 0.01; ***P < 0.001; ****P < 0.0001

Considering the effect of D594A mutation on tumor microenvironment reprogramming, we wondered whether D594A tumor respond to PD-L1 blockade in vivo. Anti-PD-L1 mAb was thus injected intraperitoneally in D594A mutant and WT MC38 tumor-bearing mice. At dissection, anti-PD-L1 treatment significantly decreased the tumor growth in both D594A and WT groups (Fig. 8A, B). Although the proportions of infiltrated CD3^+^, Macrophage and DCs populations were not altered significantly after PD-L1 treatment between D594A and WT groups, the frequency of CD8^+^ T cells was strikingly increased, with lower expression of PD-1 and TIM3 in D594A tumor (Fig. 8C).

In conclusion, D594A mutation in CRC can remodel the TIME, including upregulated the expression of PD-L1 and MHC class I, and recruit increased number of CD8^+^ T cells partially via increasing THBS1 mediated CXCL9/CXCL10 release, which can enhance antitumor potency with ICIs therapy (Fig. 8D).

## Discussion

BRAF V600E mutation in CRC has been well-described with the biological, clinical and pathological characteristics [19]. However, other mutations (BRAF non-V600) have been less investigated and remains unclear.

Consistent with previous study, it is showed that V600E (class I) mutation had hyper-activated BRAF kinase, which was dramatically reduced in D594A (class III) mutation. It is well-known that MAPK signaling directly participates into cell proliferation [20] and apoptosis. Accordingly, it is established a positively direct link between the effects of MAPK and BRAF kinase activity. Our findings showed that robust proliferation and reduced apoptosis was exhibited in V600E, but D594A induced reversely. Amino acid 594 locates in the DFG (Asp-Phe-Gly) motif of conserved region-3 (CR3) domain, which is also known as the BRAF kinase domain. Mutation occurred on D594 showed none kinase activity, confirming the phenotype that we observed [2]*.*

We found D594A was relatively sensitive to BRAFi and MEKi, established distinct biological performance from BRAF V600E and WT CRC. BRAF inhibitor (BRAFi) alone display limited clinical benefit in CRC patients with BRAF V600E mutation [21]. A combination of doublet-therapy (BRAFi and EGFR mab) or triplet-therapy (BRAFi, EGFR mab, and MEKi) indeed significantly improved the OS and response rate of mCRC patients with V600E mutation [22]. Limited data are available about the class II and III mutations. mCRC with class II mutants rarely response to EGFR antibody, while class III mutants responded extensively [23].

In this study, we observed that IFN-γ had dual effects on MC38 cells [24]. On the one hand, IFN-γ protected tumor cells from immune cell attacking by inducing PD-L1. On the other hand, IFN-γ enhanced antigen presentation capacity by upregulating the expression of MHC class I. Notably, PD-L1 and MHC class I were strongly induced in D594A compared with other mutant cells. The high expression of PD-L1 was one of the mechanisms that mediates tumor escape from immunological surveillance, it may also indicate that the activation of immunosuppressive pathway is a result of the immune excitation [25]. However, we didn’t prove the source of IFN-γ in vivo, due to the limited lymphocytes harvested from tumor.

CD8^+^ T cells exert the tumor eradication through the production of cytotoxic molecules, including perforin, granzyme, IFN-γ and TNF-α [26]. We found that CD8^+^ T cells retained better effector function when co-cultured with supernatant from D594A mutant cells. In vivo experiments displayed a higher frequency of CD8^+^ T cells in D594A tumors, with lower expression of TIM3 and PD-1, which are the co-inhibitory molecules and indicating terminally exhaustion of CD8^+^ T cells. These findings suggest that effective suppression of D594A mutant tumor rely largely on the infiltration and function of CD8^+^ T cells in the TIME.

To further explore the mechanism driving the infiltration of CD8^+^ T cells, we performed RNA-seq and identified THBS1 as an important candidate related to chemotaxis. THBS1 (thrombospondins 1) is a multifunctional matricellular protein and was first described as an angiogenesis inhibitor in 1990. The role of THBS1 in CRC is multifaceted and controversial. Maeda et al*.* thought that THBS1 was a negative-regulator of tumor angiogenesis and correlated with good prognosis [27], while Liu et al*.* proved that THBS1 was significantly correlated with liver metastasis and poor prognosis of CRC [28]. In the recent study, THBS1 was emerged to be participated into immunomodulation. THBS1 promoted CD4^+^ T cell adhesion and chemotaxis by binding to α4β1 integrin [29]. THBS1 enhanced antitumor immunity by recruiting and activating M1 typed macrophages [30]. THBS1 also inhibited NK cell proliferation by binding to CD47 [31]. However, the role of THBS1 in recruiting CD8^+^ T cells had been reported rarely. But THBS1 knockdown indirectly increased CD8^+^ TILs by increasing tumor vascularization in triple-negative breast cancer [32].

We found that THBS1 is highly expressed in cells with D594A mutation. Dual-luciferase reporter system validated that ATF3 could regulate the expression of *THBS1* by binding to its promoter. ATF3 has also been proved to drive the transcription of *PD-L1* and can be used as a biomarker for predicting the efficacy of PD-1 checkpoint blockade therapy [33]. Consistently, we noted that the expression of *ATF3* and *PD-L1* were the highest in D594A in our study. However, the correlation of ATF3 with the expression of THBS1 haven’t been explored further.

It is well recognized that the accumulation of CD8^+^ T cells was largely driven by the CXCL9/CXCL10-CXCR3 axis [18]. We found that the expression and secretion of CXCL9 and CXCL10 were higher in D594A mutant cells. Consistently, the secretion of both CXCL9 and CXCL10 were IFN-γ-dependent in MC38-bearing mice [34]. We further verified that THBS1 could promote the expression and secretion of CXCL9 and CXCL10 upon IFN-γ treatment and enhanced the migration of CD8^+^ T cells via CXCL9 and CXCL10. CXCL10 not only recruits CD8^+^ T cells, but also regulates its differentiation during chronic infection [35], while CXCL10 ablation didn’t affect the effector function of CD8^+^ T cells in this study. However, the molecular mechanism underlying the improved CD8^+^ T cell function in the TIME of D594A tumor needs further in-depth investigations.

PD-L1 is a promising drug target in cancer therapy. PD-L1 inhibitors including atezolizumab, avelumab and durvalumab were recently approved by United States Food and Drug Administration (FDA) for treating diverse types of cancer [36, 37]. MC38 cells harbor wild-type Kras and are moderately responsive to anti-PD-1 treatment in vivo [17, 38]. In view of the high expression of PD-L1 and the frequency of CD8^+^ T cells, D594A mutant CRC responded to anti-PD-L1 treatment efficiently. We surprisingly found that the infiltrated CD8^+^ T cells in D594A tumor expressing lower PD-1 and TIM3 after anti-PD-1 treatment, which predicted a better prognosis to some extent. Collectively, the effector function of CD8^+^ T cells may be more essential compared to its abundance, but the infiltration of CD8^+^ T cells was indeed more abundant in D594A mutant CRC anyway. D594A mutant CRC may be classified as “immunological tolerance type” in the CMS system, which is the ideal type to respond to immune therapy [39]. Taube et al*.* proposed that PD-L1 expression was associated with CD8^+^ T cell infiltration in Melanoma. Blocking PD-L1/PD-1 pathway may benefit patients with PD-L1^+^ tumors [40].

However, MC38 is an MSI-H model and may not accurately reflect the impacts of BRAF D594A mutation in MSS CRC [41]. In addition, our observations are based on animal experiments and it remains unclear whether it is reproducible in patients with or without mismatch repair-deficient.

## Conclusions

In total, D594A mutation exhibits lower aggressiveness among the three mutant types. Tumors with D594A mutation can be defined as “hot tumor” [42], and therefore well responsive to anti-PD-L1 treatment. The infiltration of functional CD8^+^ T cells was, at least in part, mediated by ATF3-THBS1-CXCL9/CXCL10 axis in the TIME of D594A.

### Supplementary Information

**Additional file 1: Fig. S1.** **A** Validation of mutation sites in MC38 cells with different BRAF mutations by sequencing. V600E, T1799A; G469V, G1406T; and D594A, A1781C. **B** Quantification of the bands intensity towards the blots in Fig. 1B, exploring the EGF responsiveness of the four kinds of cell lines. **C** Detection of the sensitivity of BRAF WT and mutant cells to MAPK inhibitors. Sorafenib, BRAF inhibitor; Trametinib, MEK1/2 inhibitor; SCH772984, ERK1/2 inhibitor. Three independent replicates were performed for above experiments.

**Additional file 2: Fig. S2**. Effects of different BRAF mutations on MC38 tumor growth in BALB/C nude mice (n = 8 mice per group). The tumor sizes, growth curves and weights of the tumors were shown as (**A**–**C**), respectively. Actin serves as an internal reference. *P < 0.05; **P < 0.01.

**Additional file 3: Fig. S3**. The mRNA levels of representative chemotaxis genes, *THBS1*, *B2M*, *CCL9*, *IFLH1*, *C3*, *CPL3A1*, *HFE* and *IL4RA* in different mutant cells analyzed by qRT-PCR. Three independent replicates were performed. *P < 0.05; **P < 0.01; ***P < 0.001; ****P < 0.0001.

**Additional file 4: Fig. S4**. Establishment of THBS1 overexpressing and knockdown cells. **A**, **B** The validation of THBS1-overexpression on mRNA and protein levels by qRT-PCR and western blot, respectively. hTHBS1 and hctrl cells were established based on MC38 cells. **C**, **D** The validation of THBS1-knockdown on mRNA and protein levels by qRT-PCR and western blot, respectively. siTHBS1 and sictrl cells were established based on BRAF D594A mutant MC38 cells. *P < 0.05; ****P < 0.0001.

## Data Availability

The data used to support the findings of this study are included within the article, or available from the corresponding author upon request.

## Abbreviations

CRC: Colorectal cancer
MAPK: Mitogen-activated protein kinase
BRAF: V-raf murine sarcoma viral oncogene homolog B1
MEK: Mitogen-activated extracellular signal-regulated kinase
ERK: Extracellular regulated protein kinases
THBS1: Thrombospondin 1
OS: Overall survival
PFS: Progression free survival
TIME: Tumor immune microenvironment
CXCL9: C-X-C motif ligand 9
CXCL10: C-X-C motif ligand 10
ICIs: Immune checkpoint inhibitors
TCGA: The Cancer Genome Atlas
GEO: Gene Expression Omnibus
WT: Wild type
TILs: Tumor-infiltrating lymphocytes
dMMR: Mismatch repair-deficiency
ATF3: Activating transcription factor 3
IFN-γ: Interferon gamma
TNF-α: Tumor necrosis factor alpha
ATCC: American Type Culture Collection
STR: Short tandem repeat
FBS: Fetal bovine serum
mAb: Monoclonal antibody
NK: Natural killer
DCs: Dendritic cells
MHC: Major histocompatibility complex
PD-L1: Programmed death-ligand 1
PD-L2: Programmed death-ligand-2
CCK-8: Cell counting kit-8
RNA-seq: RNA-sequencing
DEGs: Differential expressed genes
IHC: Immunohistochemistry
DFG: Asp-Phe-Gly motif
CR3: Conserved region-3
