# Supplementary figures and images for: BRAF D594A mutation defines a unique biological and immuno-modulatory subgroup associated with functional CD8+ T cell infiltration in colorectal cancer
Source: J Transl Med. 2023 Oct 18;21:737. doi: 10.1186/s12967-023-04606-5 (PMC10585750; doi:10.1186/s12967-023-04606-5)

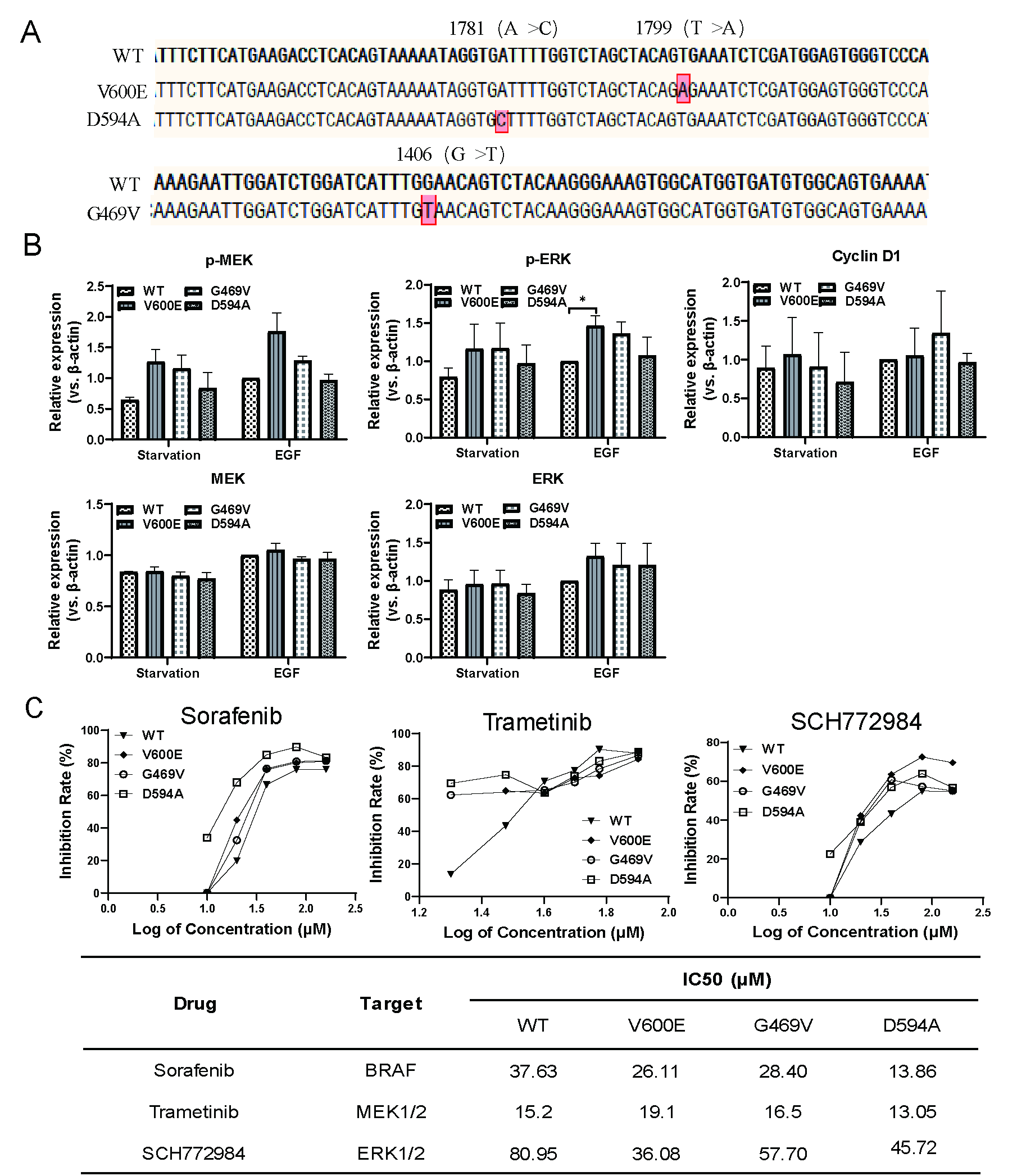

Supplement: Supplementary file 1 — Additional file 1: Fig. S1. A Validation of mutation sites in MC38 cells with different BRAF mutations by sequencing. V600E, T1799A; G469V, G1406T; and D594A, A1781C. B Quantification of the bands intensity towards the blots in Fig. 1B, exploring the EGF responsiveness of the four kinds of cell lines. C Detection of the sensitivity of BRAF WT and mutant cells to MAPK inhibitors. Sorafenib, BRAF inhibitor; Trametinib, MEK1/2 inhibitor; SCH772984, ERK1/2 inhibitor. Three independent replicates were performed for above experiments. [file 12967_2023_4606_MOESM1_ESM.tif]

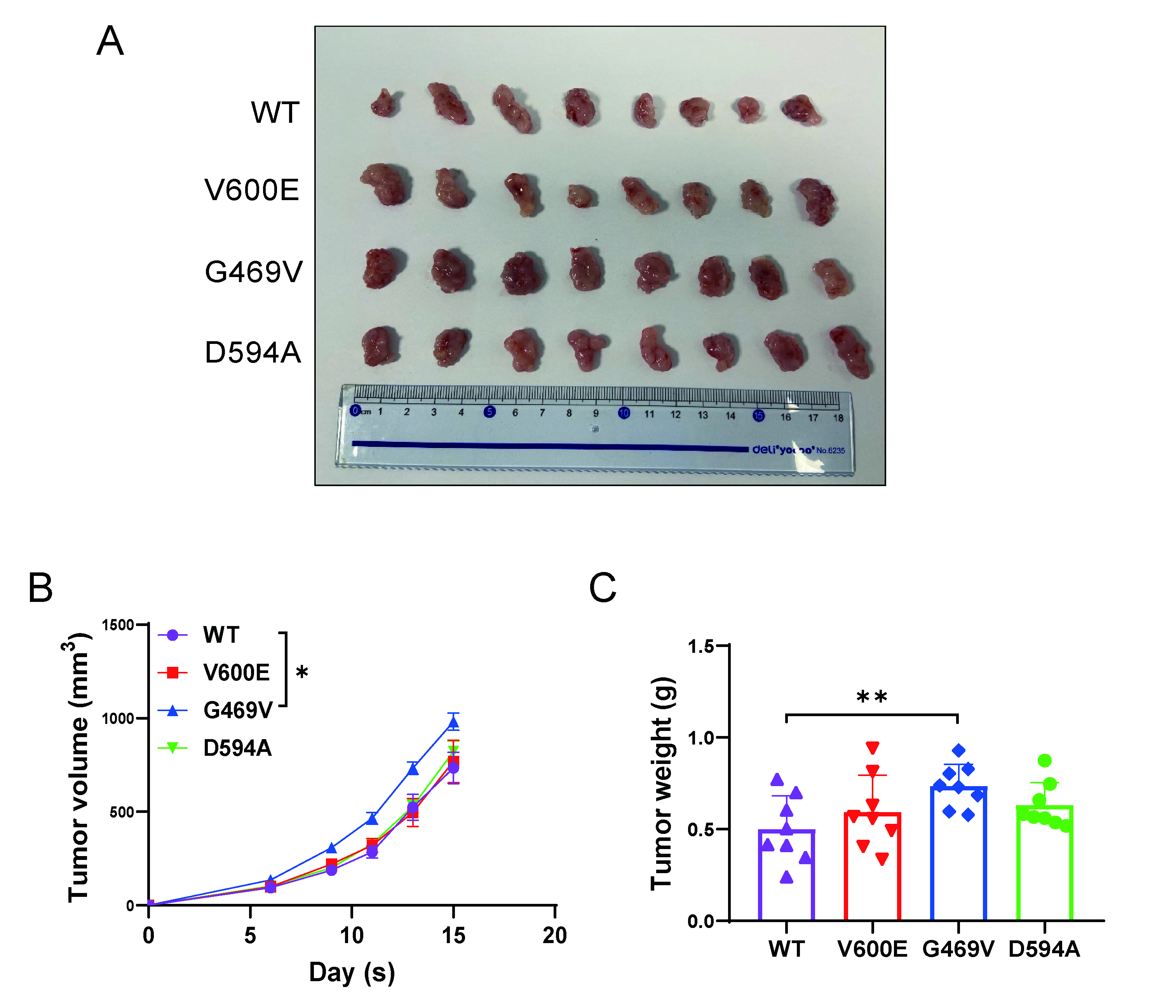

Supplement: Supplementary file 2 — Additional file 2: Fig. S2. Effects of different BRAF mutations on MC38 tumor growth in BALB/C nude mice (n = 8 mice per group). The tumor sizes, growth curves and weights of the tumors were shown as (A–C), respectively. Actin serves as an internal reference. *P < 0.05; **P < 0.01. [file 12967_2023_4606_MOESM2_ESM.tif]

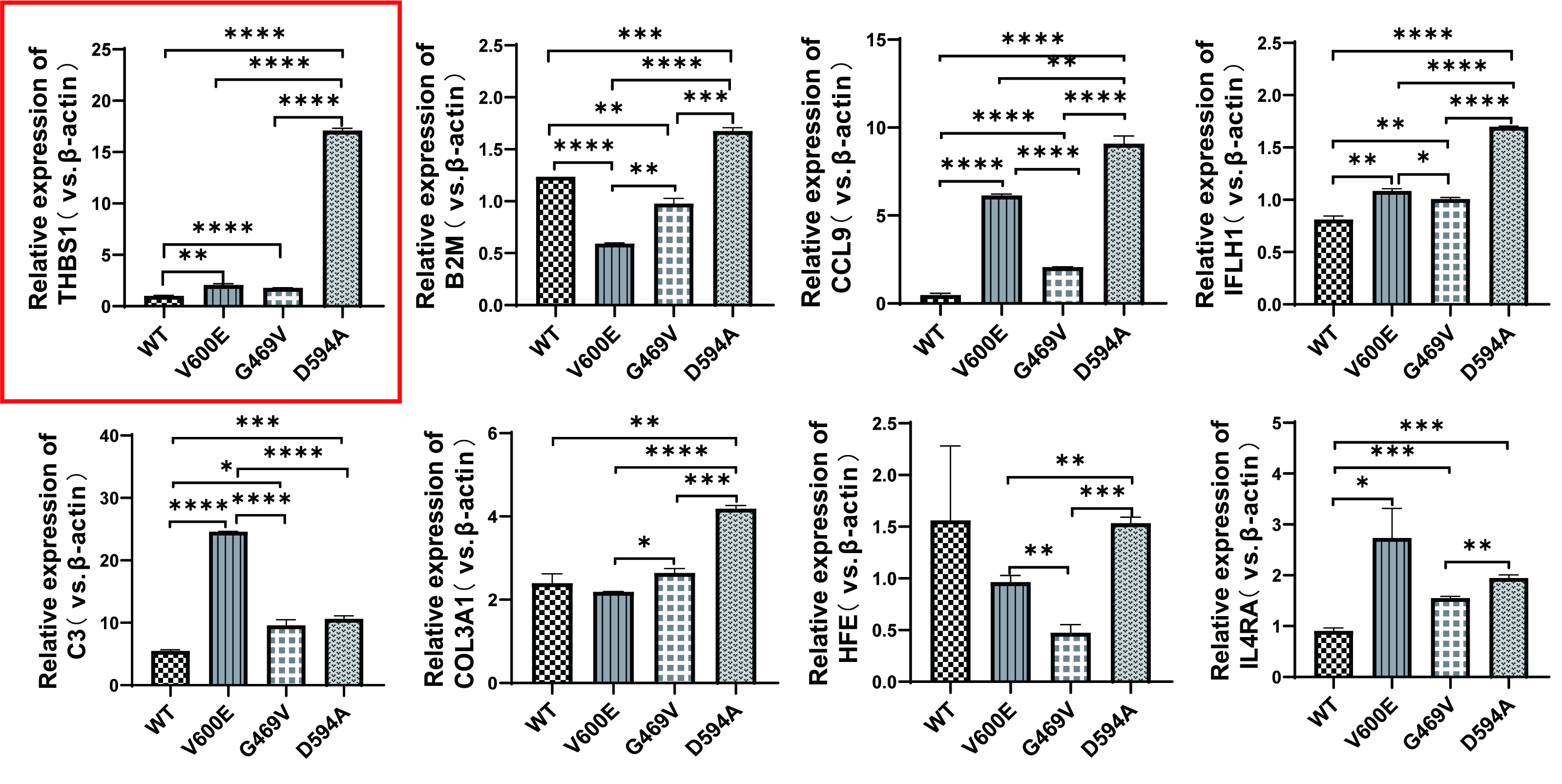

Supplement: Supplementary file 3 — Additional file 3: Fig. S3. The mRNA levels of representative chemotaxis genes, THBS1, B2M, CCL9, IFLH1, C3, CPL3A1, HFE and IL4RA in different mutant cells analyzed by qRT-PCR. Three independent replicates were performed. *P < 0.05; **P < 0.01; ***P < 0.001; ****P < 0.0001. [file 12967_2023_4606_MOESM3_ESM.tif]

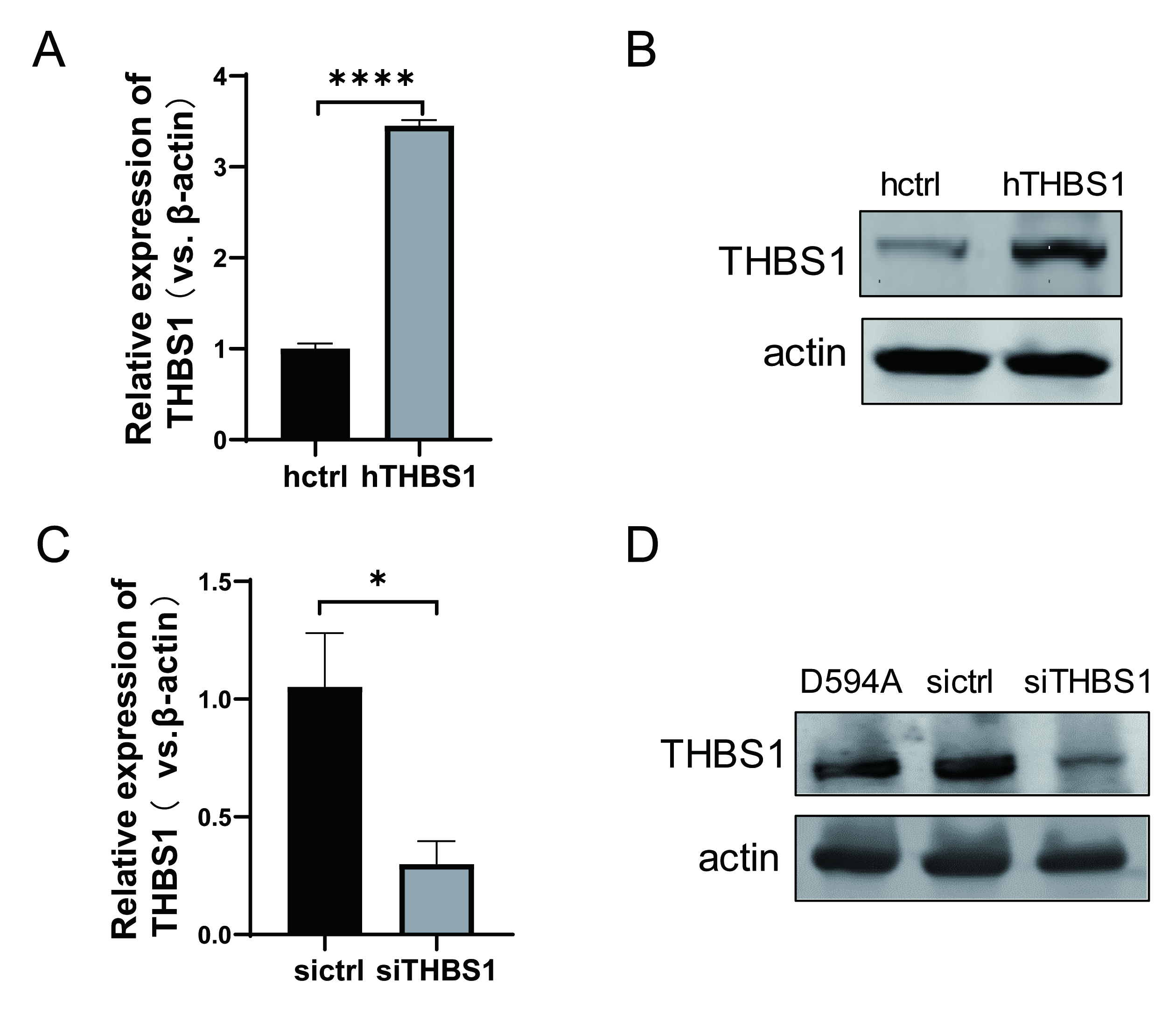

Supplement: Supplementary file 4 — Additional file 4: Fig. S4. Establishment of THBS1 overexpressing and knockdown cells. A, B The validation of THBS1-overexpression on mRNA and protein levels by qRT-PCR and western blot, respectively. hTHBS1 and hctrl cells were established based on MC38 cells. C, D The validation of THBS1-knockdown on mRNA and protein levels by qRT-PCR and western blot, respectively. siTHBS1 and sictrl cells were established based on BRAF D594A mutant MC38 cells. *P < 0.05; ****P < 0.0001. [file 12967_2023_4606_MOESM4_ESM.tif]
